# Jagged-1/Notch Pathway and Key Transient Markers Involved in Biliary Fibrosis during *Opisthorchis felineus* Infection

**DOI:** 10.3390/tropicalmed7110364

**Published:** 2022-11-09

**Authors:** Anna Kovner, Oxana Zaparina, Yaroslav Kapushchak, Galina Minkova, Viatcheslav Mordvinov, Maria Pakharukova

**Affiliations:** 1 Institute of Cytology and Genetics, Siberian Branch of the Russian Academy of Sciences, Novosibirsk 630090, Russia; 2 Institute of Molecular Biology and Biophysics, Subdivision of FRC FTM, Siberian Branch of the Russian Academy of Sciences, Novosibirsk 630117, Russia

**Keywords:** *Opisthorchis felineus*, liver fibrosis, extracellular matrix, Jagged1/Notch pathway, Syrian hamster, human

## Abstract

Chronic opisthorchiasis associated with *Opisthorchis felineus* infection is accompanied by severe fibrotic complications. It is of high practical significance to elucidate the mechanisms of hepatic fibrosis in chronic infection dynamics. The goal of the study is to investigate the temporal profile of key markers and the Jagged1/Notch signaling pathway in the implementation of fibrosis in a chronic *O. felineus* infection. For the first time, using histological methods and real-time PCR analysis, we demonstrated the activation of the Jagged1/Notch pathway in liver fibrogenesis, including the activation of the *Hes1* and *Hey1* target genes during experimental opisthorchiasis in *Mesocricetus auratus*. Cluster analysis followed by regression analysis of key markers during the infection showed that Jagged1 and Mmp9have the greatest contribution to the development of cholangiofibrosis and periductal fibrosis. Moreover, we detected a significant increase in the number of Jagged1-positive cells in the liver of chronic opisthorchiasis patients compared to that of the control group without infection. The results of the study are extremely informative both in terms of investigation both diverse fibrosis mechanisms as well as potential targets in complex antihelmintic therapy.

## 1. Introduction

*Opisthorchis felineus* belongs to a class of flatworms Trematoda (flukes). *O. felineus* causes opisthorchiasis in fish-eating mammals in wide geographical range from Eastern Europe to Central Asia, being an endemic of some regions of the Russian Federation [1]. Chronic opisthorchiasis is associated with several hepatobiliary complications common to animals and humans: cholangitis, biliary hyperplasia, hepatomegaly, periductal fibrosis and cholangiofibrosis [2,3,4,5]. In chronic opisthorchiasis, the progression of fibrotic implications is associated with several factors: (i) mechanical damage of the bile ducts by *O. felineus* [4,6]; (ii) high levels of oxidative stress [7,8]; (iii) *O. felineus*-induced host inflammation [4,9]. Remarkably, fibrosis is one of the critical contributing factors to the opisthorchiasis complication [10].

It has been shown that myofibroblasts are the cells that are ultimately responsible for the severe outcome of the fibrotic process [11]. Myofibroblasts can originate from various sources of fibroblasts and liver stellate cells [12]. Myofibroblasts begin to express α-smooth muscle actin (α-SMA) and collagen with the formation of excess extracellular matrix (ECM), which leads to liver fibrosis [13]. Moreover, the epithelial-to-mesenchymal transition (EMT) may be one of the potential mechanisms for the formation of myofibroblast-like cells [14,15]. The biological meaning of the process is reduced to the loss of the epithelial profile (E-cadherin) and acquisition of a mesenchymal-like profile (N-cadherin, vimentin). However, data on EMT remains controversial, and its role in liver fibrogenesis accompanied by chronic opisthorchiasis is poorly understood [16].

One of the main signaling pathways implementing EMT is the Jagged1/Notch pathway [14]. This cascade is activated in many models of liver fibrosis and is abnormally increased in patients with fibrotic complications in several diseases, including non-alcoholic steatohepatitis NASH [17,18,19]. It was also shown that the Jagged1/Notch signaling pathway could selectively mediate the fibrogenic properties of TGFβ1, which is required to stimulate the production and deposition of ECM components [20]. Notch-activated hepatocytes also contributed to severe liver fibrosis due to additional HSC activation in vitro and in vivo [21]. In a mouse model, it was also shown that *Schistosoma japonicum* infection caused Jagged1/Notch1 signaling pathway activation, which in turn stimulated the macrophages polarization along the profibrotic M2 phenotype [22].

It is known that the treatment of opisthorchiasis in humans often improves the gallbladder condition [23]. However, monitoring patients after praziquantel treatment demonstrated persistence or recurrence of periductal liver fibrosis [24,25]. This dictates an urgent need to scrutinize processes of fibrogenesis accompanied by chronic opisthorchiasis in order to search for new promising anti-fibrotic molecular targets. Thus, the aim of the study was to evaluate the dynamics of effector cells changing, various pathways and especially the Jagged1/Notch pathway in liver fibrogenesis in Syrian hamster model and also to detect Jagged1 in humans with chronic liver damage, including *O. felineus* infection.

## 2. Materials and Methods

### 2.1. Ethical Statement

All the procedures are in compliance with EU Directive 2010/63/EU for animal experiments and in compliance with The Code of Ethics of the World Medical Association (Declaration of Helsinki) for human study. Study design protocols and standard operating procedures were approved by the Ethical Committee for Animal Experiments at the ICG SB RAS (permit number 25 from 12 December 2014 (approved for hamsters and fish)).

The human biological samples and further obtaining data were approved by the Ethics Committee at the Institute of Molecular Biology and Biophysics, SB RAS (permit number 2/2016 from 27 October 2016). All the participants provided written informed consent. Participant age ranged from 40 to 81 years. Liver samples from people with and without opisthorchiasis were used in the experiment [5].

### 2.2. Parasites, Hamsters, and Experimental Design

Liver fluke metacercariae were collected from naturally infected *Leuciscus idus* (the Ob River, Novosibirsk, Western Siberia) and isolated via standard protocol in accordance with all regulations of the Russian Federation [5]. Male Syrian hamsters aged 6–8 weeks were obtained from the Conventional Animal Facility of the Institute of Cytology and Genetics (Novosibirsk, Russia) and were orally infected with 50 *O. felineus* metacercariae. Rodents were kept in standard conditions on a normal diet and free access to water. Control non-infected hamsters (n = 20) and *O. felineus*-infected hamsters (n = 40) were euthanized using carbon dioxide and withdrawn 10, 14, 18, 22, 26, 30, 34, and 52 weeks after the infection.

The four-stage blinding study protocol was applied: first, researchers divided the groups of animals based on randomization; second, researchers monitored the animals and administered anesthesia; third, researchers performed all surgical interventions and selected material for research; fourth, researchers described morphological changes and analyzed gene expression.

### 2.3. Sample Collection

In hamsters, tissue samples were taken from the large right lobe of the liver and (i) were placed into RNAlater (ThermoFisher Scientific, Waltham, MA, USA); (ii) were fixed in a 10% aqueous solution of neutral formalin and were dehydrated in a graded series of ethanol and in xylene (STP-120, Thermo Scientific). Dehydrated samples were enclosed in a paraffinic medium HISTOMIX (BioVitrum, Saint Petersburg, Russia). For microscopic examination, sections of 3.5 μm thickness were prepared on a rotary microtome Microm HM 355S (ThermoFisher Scientific, Waltham, MA, USA). Human gallbladder tissue samples along with liver samples were subjected to similar chemical processing.

### 2.4. Histopathology and Immunohistochemistry

The resulting paraffin sections were stained via a standard protocol with hematoxylin and eosin and Van Gieson’s staining (detecting connective tissue fibers). To determine the phenotype of effector cells, typing and assessment of collagen ECM, EMT definition was carried out using an immunohistochemical kit (SpringBioScience kit HRP-125) and specific primary antibodies:

(1) The extracellular matrix state: collagen I (Abcam, cat. # ab34710, 1:200), Mmp2 (SpringBioScience, E18014, 1:100), Mmp9 (Abcam, ab58803, 1:100), TIMP1 (Abcam, ab216432, 1:100);
(2) Fibrosis regulation system: transforming growth factor (TGFβ1; Abcam, ab92486, 1:200), SMAD2 (Abcam, ab219598; 1:250), α-smooth muscle actin (α-SMA; Abcam, ab7817, 1:300), Jagged1 (Cloud-Clone, PAB807Mu01, 1:100);
(3) Effector cell markers: fibroblast surface protein FSP (Abcam, ab11333, 1:300), glial fibrillary acidic protein (GFAP) from hepatic stellate cells (HSCs) (Abcam, ab7260, 1:300), E-cadherin (Abcam, ab 76055, 1:500), vimentin (Abcam, ab 8069, 1:200).

Staining was performed according to the manufacturer’s protocol. Visualization was carried out under an AxioImager A1 microscope (Zeiss) with camera AxioCam MRc (Zeiss).

To assess the state of cholangiofibrosis, 20 random visual fields of parenchyma were used. We also evaluated periductal fibrosis in 10 visual fields in the hepatic large bile duct in a closed test system 3.6 × 10^5^ μm^2^ per 100 points. In a closed test grid system per 100 points, the number of positive cells and the percentage of connective tissue intersection with the grid were analyzed. The calculation protocol has been previously standardized and tested [9].

### 2.5. RT-PCR

Total RNA was isolated using the ExtractRNA (Evrogen, Moscow, Russia). Concentrations of RNA were measured using a NanoDrop spectrophotometer (ND1000, NanoDrop Technologies, Wilmington, DE, USA). First-strand cDNA synthesis was performed with RevertAid Kit (Fermentas, European Union). Gene expression levels were measured using real-time PCR with the EVA Green Reagent Mix (Synthol, Moscow, Russia) on a CFX96 real-time PCR system (Bio-Rad, Hercules, CA, USA). The Gapdh gene was chosen as an endogenous internal control. Triplicate real-time PCRs were performed for each sample. The fold-change in target gene expression (normalized to the controls) was calculated from threshold cycle values (Ct; CFX96 software). Sequences for all primers and probes can be found in Table S1 (Synthol, Moscow, Russia).

### 2.6. Statistical Analysis

The data were analyzed using Statistica 6.0 software (Statsoft, Tulsa, OK, USA) and R customized scripts (4.1.0, “ggplot2”, “gplots” packages). Some of the variables have been transformed using standard methods to better fit the normal distribution (log10—transformation and sqrt—transformation). Differences between control and experimental groups were assessed using ANOVA + Newman–Keuls test (for normally distributed data) and Kruskal–Wallis + paired Wilcoxon test (non-normally distributed data). Using one-way linear regression analysis, we assessed the dependence of parameters on the duration of infection. Clusters of parameters were identified that changed in a similar manner during the course of the infection using the heatmap function.2 (gplots package). Obtained parameters were used to build multiple linear regression models, where periductal and cholangiofibrosis were used as dependent variables. Linear models were built in R using a standard package.

## 3. Results

### 3.1. Liver Fibrosis in the Course of Opisthorchis felineus Infection

**Figure 1:**
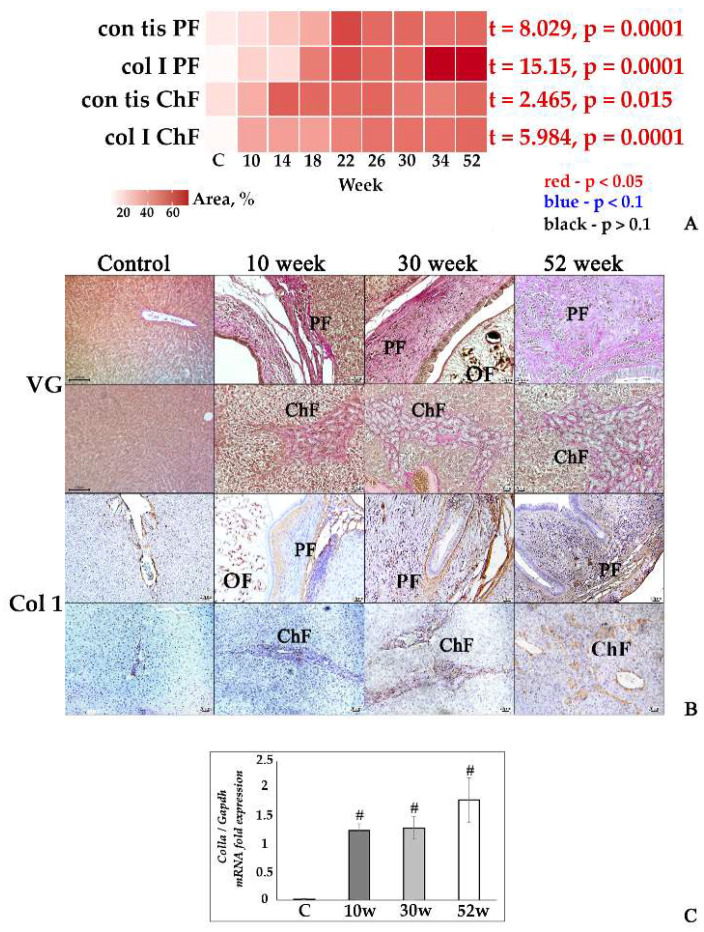
Fibrotic changes in the liver tissue of *O. felineus*-infected Syrian hamsters. (**A**) Heat map progression of periductal fibrosis (con_tis_PF) and cholangiofibrosis (con_tis_ChF), and the amount of collagen 1a+ fibers in the liver of infected animals (col_I_PF and col_I_ChF, respectively); (**B**) histopathological changes in Syrian hamster liver, Van Gieson staining (VG), and IHC analysis for collagen 1a (Col1); (C) *Col1a* gene was normalized to average *Gapdh* expression. C—control, 10 w, 30 w, 52 w—week p.i. Data are presented as mean ± SEM, # *p* ≤ 0.05, as compared to the control group.

Considerable progression of both periductal and cholangiofibrosis with a significant amount of type 1 collagen deposition was detected in the liver of golden hamsters with the increasing longevity of *Opisthorchis felineus* infection. In uninfected control hamsters, a slight deposition of connective tissue was detected, which corresponds to the norm (Figure 1A,B; Figure S2). The expression of the *Col1a* gene in the liver of infected hamsters was increased relative to the control at all experimental time points. However, there were no significant differences between infection time points (Figure 1C). It should also be noted that no significant differences were found in control animals at different experimental time points. In this regard, all control animals were combined into a common group.

**Figure 2:**
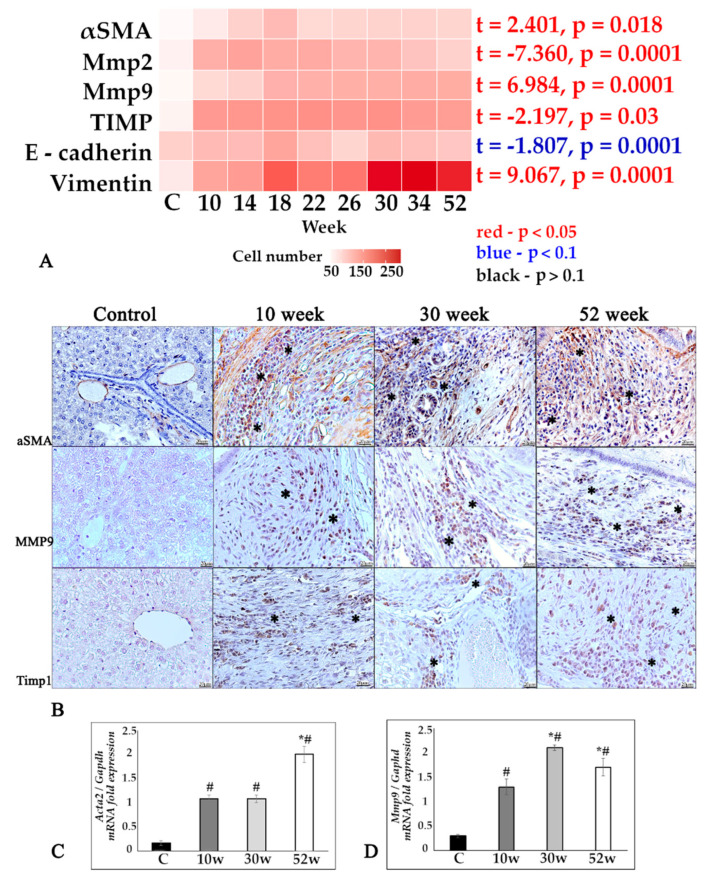
State of extracellular matrix in chronic opisthorchiasis, associated with *O. felineus* infection in the Syrian hamster model. (**A**) A heat map of ECM component changes; (**B**) IHC study of α-SMA-, Mmp9-, and TIMP1-positive cells (marked by an asterisk) in study dynamics, magnification ×400; (**C**,**D**) *Acta2*, *Mmp9* were normalized to average *Gapdh* expression. C—control, 10 w, 30 w, 52 w—week p.i. Data are presented as mean ± SEM, # *p* ≤ 0.05, as compared to the control group, * *p* ≤ 0.05, as compared to the previous study period.

The ECM state was also accompanied by an assessment of α-smooth muscle actin, reflecting myofibroblasts activation. The number of α-SMA-positive cells significantly increased during the experiment (Figure 2A,B). Large number of αSMA+ fibers was also detected in a cholangiofibrosis and periductal fibrosis areas (Figure 2B). This was accompanied by the *Acta2* gene activation at all stages of infection, with a significant increase at week 52, which may indicate the ongoing activation of fibroblastic processes even at such late stages of infection (Figure 2C).

The state of matrix metalloproteinases (Mmp2, 9) and their tissue inhibitor (TIMP1) was assessed. During the progression of chronic opisthorchiasis, the number of Mmp2+, Mmp9+, and TIMP1+ cells in the liver of infected hamsters significantly increased (Figure 2A). The *Mmp9* gene expression was increased at all study periods relative to the control, with a peak at the 30th week (Figure 2D). It should also be noted that the number of E-cadherin+ cells did not significantly change during the experiment, while the number of vimentin+ cells significantly increased (Figure 2A).

**Figure 3:**
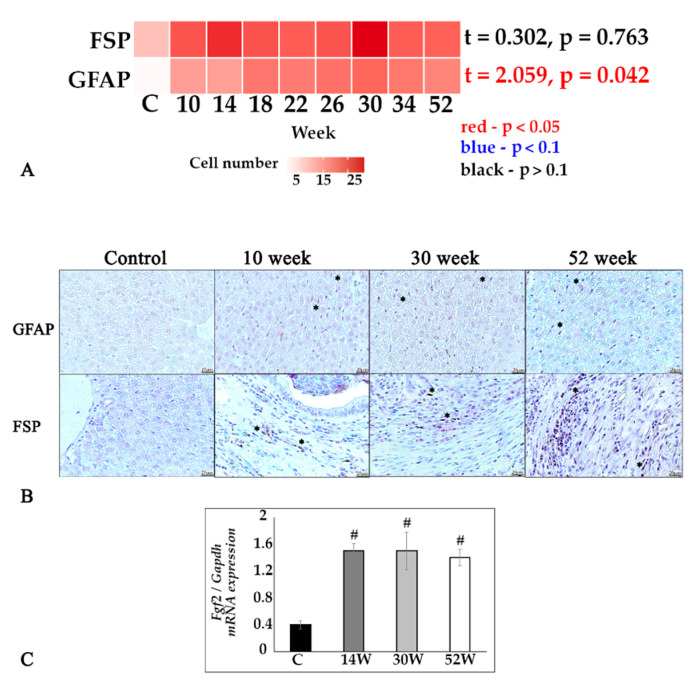
Stellate cells and fibroblasts in the liver tissue of *O. felineus* -infected Syrian hamsters. (**A**) Heat map of changes of FSP- and GFAP-positive cell numbers in the dynamics of infection; (**B**) GFAP- and FSP-positive cells (marked by an asterisk), IHC study, ×400 magnification; (**C**) *Fgf2* gene was normalized to average *Gapdh* expression. C—control, 10 w, 30 w, 52 w—week p.i. Data are presented as mean ± SEM, # *p* ≤ 0.05, as compared to the control group.

The number of FSP-positive fibroblasts did not significantly depend on the experiment duration (Figure 3A,B). The number of GFAP-positive hepatic stellate cells significantly increased during the course of the infection (Figure 3A,B). Analysis of the *Fgf2* gene expression (fibroblast growth factor) also showed no significant changes between the periods of the study, with a significant difference compared to the control group (Figure 3C).

### 3.2. Signaling Pathways Associated with Chronic Opisthorchiasis in Syrian Hamsters

**Figure 4:**
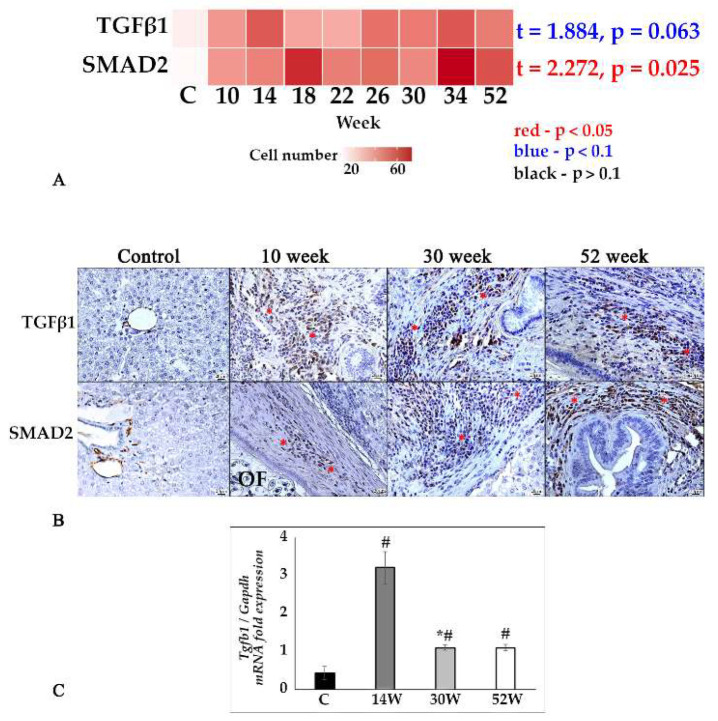
TGFβ1/SMAD2 signaling pathway in the liver tissue of *O. felineus*-infected Syrian hamsters. (**A**) Heat map of changes in TGF-β1- and SMAD2-positive cells in the course of infection; (**B**) TGF-β1- and SMAD2-positive cells (marked by an asterisk) in the dynamics of infection, IHC study, ×400 magnification; (**C**) *Tgfb1* gene was normalized to average *Gapdh* expression. C—control, 10 w, 30 w, 52 w—week p.i. Data are presented as mean ± SEM, # *p* ≤ 0.05, as compared to the control group, * *p* ≤ 0.05, as compared to the previous period of investigation.

TGFβ1 and SMAD 2/3 are key activators and mediators of fibrogenesis. A quantitative analysis of histological sections showed that the TGFβ1-positive cell number was increased during all stages of the experiment (from 10 to 52 weeks) without reaching significant changes at the trend level. The peak of the *Tgfb1* gene expression was at the 10th week, with further significant decrease by the 30th week p.i. At all periods of the experiment, the expression of this gene significantly exceeded the respective control values (Figure 4A–C). However, quantitative analysis of histological samples of SMAD2-positive hepatic cells significantly increased during the course of infection (Figure 4A,B).

**Figure 5:**
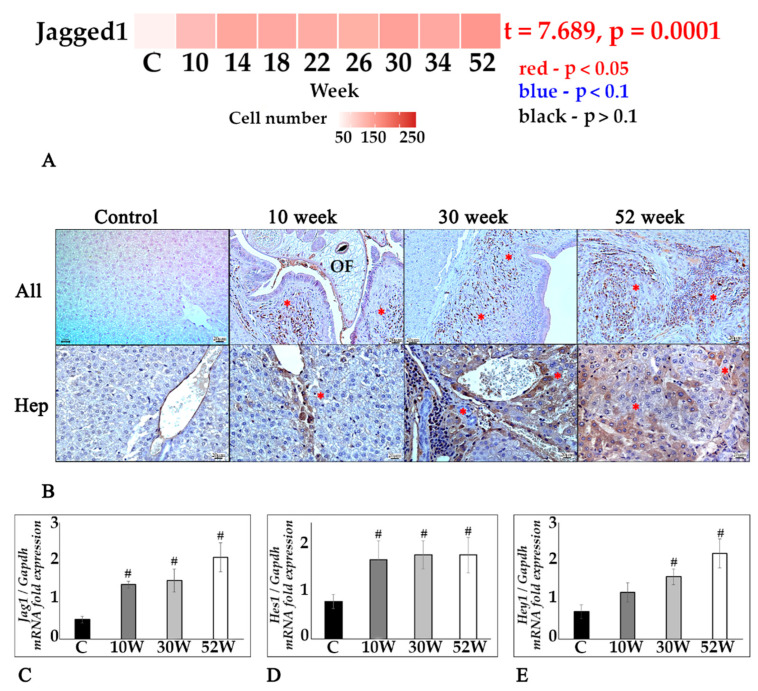
Jagged1/Notch pathway in the liver tissue in chronic opisthorchiasis in Syrian hamsters. (**A**) Heat map changes in Jagged1-positive cells in infection dynamics; (**B**) IHC staining for Jagged1, Jagged1+ cells in the area of periductal fibrosis (All) (marked by an asterisk), magnification ×200, Jagged1+hepatocytes in the area of cholangiofibrosis (Hep) (marked by an asterisk), magnification ×400; (**C**–**E**) *Jag1, Hey1, Hes1* were normalized to average *Gapdh* expression. C—control, 10 w, 30 w, 52 w—week p.i. Data are presented as mean ± SEM, # *p* ≤ 0.05, as compared to the control group.

One of the most common EMT pathways in liver fibrosis is the Notch pathway, which is characterized by the activation of its Jagged1 ligand throughout the study. IHC staining revealed an increase in the number of Jagged1-positive cells as early as week 10 of the experiment and remained high even at week 52 (Figure 5A,B). At the same time, the number of Jagged1+ hepatocytes increased during the course of the infection is localized mainly in the area of cholangiofibrosis (Figure 5B). The expression of the *Jag1* gene was also increased compared to the control at all stages of infection (Figure 5C). Notch target genes are represented by hairy and enhancers of the split (Hes) family, including *Hes1* and Hey gene family (Hes contains YRPW motif) such as *Hey1*. Thus, the activation of the Notch pathway is also reflected by the increased expression of the *Hes1* and *Hey1* genes throughout the experiment (Figure 5D,E).

### 3.3. Jagged1 Staining in Humans

**Figure 6:**
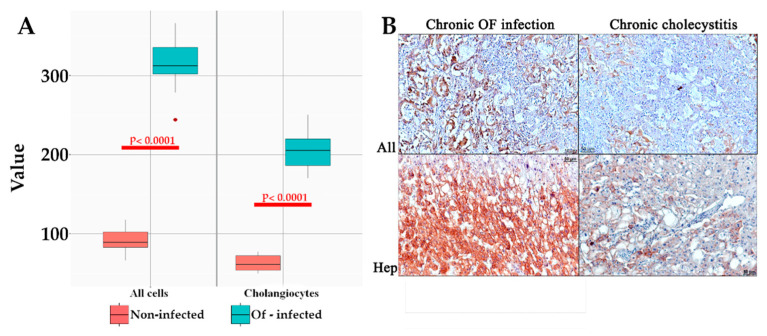
Jagged1/Notch pathway in the liver tissue in chronic opisthorchiasis and chronic cholecystitis in humans. (**A**) Boxplot of Jagged1-positive cell count data; (**B**) Jagged1-positive cells in chronic liver injury with and without *O. felineus* infection, IHC staining, magnification ×200.

Evaluating human liver sections, Jagged1+ staining was detected in both cholangiocytes and hepatocytes (Figure 6A). It is also important to note that a visual difference was found for Jagged1-positive staining: significant severity in chronic opisthorchiasis *O. felineus* and less pronounced staining in non-*O. felineus* chronic cholangitis (Figure 6A,B).

### 3.4. Cluster Analysis and Logical Regression Models of Fibrogenesis Key Factors

**Figure 7:**
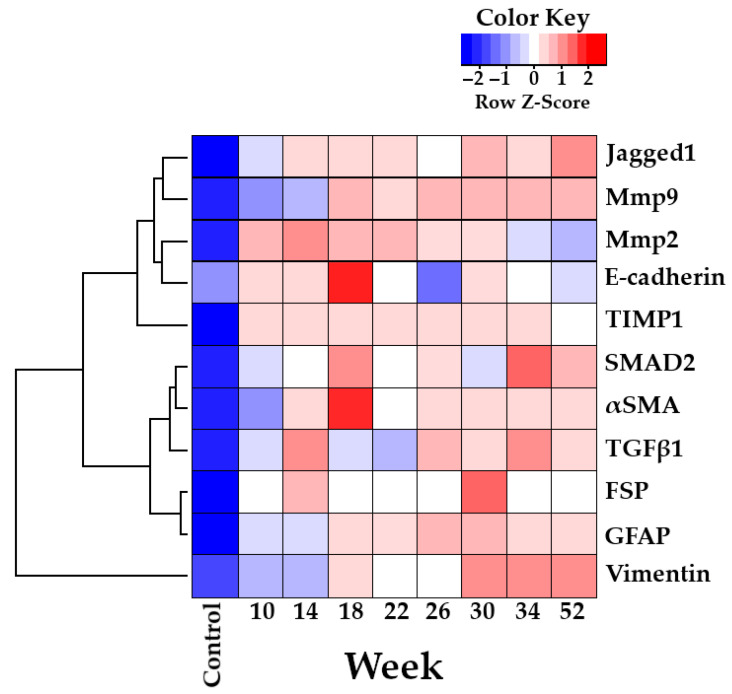
Cluster analysis of 11 markers of fibrosis (interaction of factors: “infection × time”) in hamster liver (*Mesocricetus auratus*) infected with *Opisthorchis felineus* (from 10 to 52 weeks p.i.).

Next, we were interested in an in-depth analysis of markers reflecting fibrogenesis and potential markers identifying possible signaling pathways (interaction of factors: “time × infection”) to find clusters of markers that have certain patterns in groups of liver samples. Thus, samples were divided into three large groups (Figure 7). In particular, group 1 was clustered into three clusters (Jagged1+Mmp9), (Mmp2+E-cadherin), (TIMP1); group 2 was also clustered into three clusters (SMAD2+αSMA), (Tgfβ1), (FSP+GFAP); group 3 was contained only one cluster (vimentin). The clustering of periductal and cholangiofibrosis with type I collagen is presented in Supplementary Materials Figure S2.

**Figure 8:**
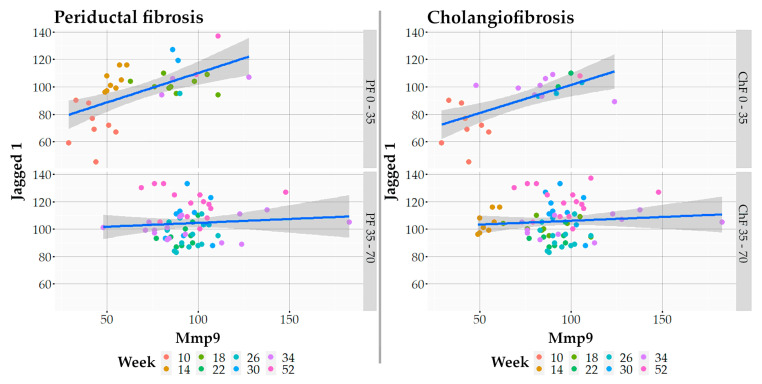
Multiple regression analysis of periductal and cholangiofibrosis Jagged1 and Mmp9 markers. Gray on the graph indicates the distribution of the standard deviation, showing the scatter of the data. Values for each week are coded with their own color.

Further, using a multiple logical regression model, we tried to assess the greatest contribution of factors on the development of chronic periductal and cholangiofibrosis in the dynamics of infection based on the obtained cluster analysis (Figure 7). As a result of constructing mathematical model no. 1 for the relationship of factors and periductal fibrosis, the greatest contribution was made by Jagged1 (t-value 3.427, Pr (>|t|) 0.001), Mmp9 (t-value 4.248, Pr (>|t|) 0.000) and their negative interaction (t-value -3.097, Pr (>|t|) 0.003) (Figure 8, Supplementary Materials S1, Table S2). Resulting from the construction of mathematical model no. 2 for the relationship of factors and cholangiofibrosis, the greatest contribution was also made by Jagged1 (t-value 5.622, Pr (>|t|) 0.000), Mmp9 (t-value 4.744, Pr (>|t|) 0.000) and their negative interaction (t-value -4.477, Pr (>|t|) 0.000) (Figure 8, Supplementary Materials S1, Table S2). Moreover, if the values of periductal fibrosis and cholangiofibrosis were less than 35% (Figure 1), then the influence of factors was increasing (Figure 8, upper line), and if the values of periductal fibrosis and cholangiofibrosis were more than 35% (Figure 1), then the influence factors were plateaued (Figure 8, lower line). Analysis of the obtained data suggested that after 22 weeks p.i. (PF > 35%, ChF > 35%), these factors became independent relative to each other.

## 4. Discussion

In this study, we demonstrated for the first time, in a model of Syrian hamsters with chronic *Opisthorchis felineus* infection, the involvement of the Jagged1/Notch signaling pathway in the progression of chronic opisthorchiasis after 50 weeks of infection. This was accompanied by the *Hes1* and *Hey1* target genes activation up to 52 weeks p.i. in Syrian hamsters. Cluster analysis with further regression analysis of fibrogenesis key markers of infection dynamics showed that Jagged1, Mmp9, as well as their interaction, have the greatest contribution to the development of cholangio- and periductal fibrosis. A significant difference was also detected in human liver samples: in chronic opisthorchiasis, the number of Jagged1-positive cells (hepatocytes, cholangiocytes, and inflammatory cells) significantly increased compared to the group without infection. Obtained data are another evidence of the suitability of Syrian hamster as an experimental model of opisthorchiasis [5].

Despite the rather successful therapy of helminthiases, more than 40% of patients with opisthorchiasis have pronounced periductal fibrosis after a 2-year follow-up. The factors causing this phenomenon remain unknown [25]. In this regard, elucidating potential long-term mechanisms of fibrogenesis during chronic opisthorchiasis seems to be highly relevant.

We have shown that for more than a year after infection with *O. felineus*: (i) amount of connective tissue increased significantly (without reaching a plateau); (ii) deposition of collagen type 1 increased with a significant increase in the expression of the *Col1a1* gene at week 52 p.i.; (iii) Mmps/TIMP imbalance was detected at all stages of infection.

Hepatic myofibroblasts are a heterogeneous population of α-smooth muscle actin (α-SMA) expressing liver cells that play a critical profibrogenic role in the progression of chronic liver disease [26]. In chronic opisthorchiasis in the liver of Syrian hamsters, the number of α-SMA+ cells significantly increases by the 52nd week of the experiment, with the peak expression of this gene at 52 weeks. This may indicate the progression of the fibrotic process even a year after infection. In chronic opisthorchiasis associated with *O. viverrini*, a high number of α-SMA+ cells was also detected [27,28].

Despite numerous studies, the main source of myofibroblasts in hepatic fibrosis remains unknown [29]. It is currently assumed that the main precursor cells are GFAP + hepatic stellate cells [30] and portal fibroblasts [31]. Significant numbers of GFAP+ cells have been identified in human livers in chronic *Schistosoma mansoni* infection [32]. However, during chronic *O. felineus* infection in experimental animals, only the number of GFAP+ cells (not FSP+ fibroblasts) significantly increased during the course of infection, remaining, nevertheless, comparatively small.

However, one possibility for hepatic stellate cell activation could be EMT via the Jagged1/Notch signaling pathway [33]. It is known that the participants of the Notch signaling pathway, including Notch ligand *Jag1* and target genes *Hes1* and *Hey1*, have been identified as genes sensitive to *Tgfb1*, which also leads to a high-level expression of myofibroblasts marker α-SMA/*Acta2* [34]. The increased *Jag1* gene expression has also been shown to be responsible for Notch pathway reactivation in the fibrosis process [18]. In particular, we noted that, as *O. felineus* infection progressed, the number of Jagged1-positive cells significantly increased, as well as the expression of the *Jag1, Hes1,* and *Hey1* genes, which reflects the activation of the Jagged1/Notch signaling pathway. In addition, using regression analysis, we identified Jagged1 as one of the key markers of cholangio- and periductal fibrosis. The obtained data are consistent with those obtained earlier. We demonstrated that EMT is the most enriched term among MSigDB hallmarks in the infected animals. The TGFβ1 pathway was also one of the most enriched terms in the set of differentially expressed genes associated with the infection [16].

Activation of the *Jag1* gene expression by hepatocytes can enhance liver fibrosis, including through the activation of hepatic stellate cells. It has also been demonstrated that the *Jag1* activation in hepatocytes reflects Notch pathway activation and is an indicator of non-alcoholic fatty liver disease in human liver biopsy specimens and in a mouse model [18]. Comparing chronic *O. felineus* infection and chronic non-*O. felineus* cholecystitis in humans, we showed for the first time a significant variation in the number of Jagged1+ cells, with an obvious predominance in infected people. Staining was detected mainly in cholangiocytes of the bile ducts, hepatocytes, and cells of the inflammatory infiltrate. In the study of liver tissues of Syrian hamsters, positive Jagged1 staining was also detected in hepatocytes, the number of which increased with the duration of infection. There are no similar data using other trematodosis models; however, it has been shown that the activation of upstream Notch signaling molecules are critical processes that stimulate cholangiocarcinoma (CCA) formation through the transformation of mature hepatocytes into CCA cells [35]. This may remind chronic infection with *Opisthorchis viverrini*, which is the major risk factor for CCA development [36]. Importantly, among the predicted miRNAs targets in 321 human and cattle messenger RNAs, the Wnt signaling pathway (other EMT signaling pathway) fibrosis-associated genes in *S. mansoni* and *F. hepatica* have particularly remarkable expression patterns [37].

Epithelial-to-mesenchymal transition (e.g., Jagged1/Notch pathway) occurrence is related to the activation of the Smad-dependent pathway and mitogen-activated protein kinase (MAPK) pathway mediated by TGFβ1 [38,39]. Interestingly, Notch signaling can directly regulate Mmp9 expression by enhancing its promoter activity, as shown in a model of pancreatic cancer and a model of wound healing in diabetes [40,41]. This is likely also characteristic of chronic liver fibrosis in the presence of *O. felineus* infection, which may explain the clustering of Jagged1 and Mmp9, as well as their predicted interaction, probably due to an imbalance in Mmps/TIMP1. Notch inhibition has previously been shown to reduce HSC activity in vitro [41,42].

*Opisthorchis felineus* has the ability to survive in host bile ducts for more than 10 years [43]. Our data demonstrated linear progression in cholangiofibrosis and periductal fibrosis values during chronic opisthorchiasis. This might be associated with various factors: (i) mechanical damage to the bile duct [9]; (ii) host defense immunological response [4]; (iii) accumulation of oxidative stress and oxidative lesions [8]; and (iv) parasite-derived excretory-secretory products [9]. Mechanical damage induced by the feeding activities of liver flukes contributes to biliary damage. As the flukes mature, the lesions enlarge, ulcerate, and lead to fibrosis progression [36]. In *O. viverrini* and *O. felineus* infection, the prevalence of type 2 (anti-inflammatory) immune response, including transforming growth factor Tgfb1 activation was demonstrated. These immunosuppressive responses may promote both *O. viverrini* infection and survival as well as host fibrotic changes [44,45]. The formation of the M2 macrophage phenotype also contributes to fibrosis development [10]. Previously, we demonstrated that *O. felineus*-associated chronic inflammation increased oxidative stress [9]. Chronic inflammation facilitates myofibroblasts activation and periductal and cholangiofibrosis. In this experiment, we have shown the linear progression of αSMA+-positive cell number. On the other hand, excretory-secretory product proteins can interact with the epithelium and accumulate inside the cells. It was shown that components of *Clonorchis sinensis* excretory-secretory products, such as secretory phospholipase A(2), fructose-1, 6-bisphosphatase, lysophospholipase, and Fe heavy chain protein, directly activated human HSCs and other key cells in hepatic fibrosis process [46]. Finally, consistent with these findings, *O. viverrini* and *O. felineus* are known to secrete glutathione S-transferase [9,47], known as EMT inducers [47]. Thus, fibrogenesis associated with chronic opisthorchiasis is a complex multi-stage process.

## 5. Conclusions

Altogether, our present investigation demonstrates that activation of Jagged1/Notch1 signaling was one of the main fibrotic mechanisms during chronic opisthorchiasis. Thus, the obtained data on the contribution of the Jagged1/Notch signaling pathway to the development of liver fibrosis accompanied by chronic opisthorchiasis *Opisthorchis felineus* are of great interest both in terms of detecting potential mechanisms of fibrosis as well as potential targets in complex anthelmintic therapy.

## Data Availability

All data generated or analyzed during this study are included in this published article.

## Supplementary Materials

The following supporting information can be downloaded at: https://www.mdpi.com/article/10.3390/tropicalmed7110364/s1, Supplementary Material S1: Table S1: Primers and probes used for real-time PCR in this study, Table S2: Multiple logical regression model demonstrated the greatest contribution of factors on the development of chronic periductal and cholangiofibrosis in the dynamics of infection. Supplementary Material S2: Figure S1: Histopathological changes in Syrian hamster liver, Figure S2: Clustering analysis of periductal and cholangiofibrosis with type I collagen. Figure S3: Calculation of parametric criterion Pearson correlation. Figure S4: Calculation of non-parametric criterion Spearman correlation.
