# Correction: Kovner et al. Jagged-1/Notch Pathway and Key Transient Markers Involved in Biliary Fibrosis during *Opisthorchis felineus* Infection. *Trop. Med. Infect. Dis.* 2022, *7*, 364

**DOI:** 10.3390/tropicalmed10100298

**Published:** 2025-10-20

**Authors:** Anna Kovner, Oxana Zaparina, Yaroslav Kapushchak, Galina Minkova, Viatcheslav Mordvinov, Maria Pakharukova

**Affiliations:** 1 Institute of Cytology and Genetics, Siberian Branch of the Russian Academy of Sciences, Novosibirsk 630090, Russia; 2 Institute of Molecular Biology and Biophysics, Subdivision of FRC FTM, Siberian Branch of the Russian Academy of Sciences, Novosibirsk 630117, Russia

In the original publication [1], there was a mistake in Figure 1 and Figure 4 as published. In Collage 1B, the original images for the ‘Col 1’ control group and the upper 30-week group in Figure 1 do not depict the bile duct. Additionally, the lower 52-week ‘Col 1’ group contained a technical error and has been replaced with a representative image. In Collage 4B, the original ‘SMAD’ control group in Figure 4 did not clearly show the positive staining in the portal triad areas. The corrected Figure 1 and Figure 4 are shown below. The authors state that the scientific conclusions are unaffected. This correction was approved by the Academic Editor. The original publication has also been updated.

## Figures and Tables

**Figure 1:**
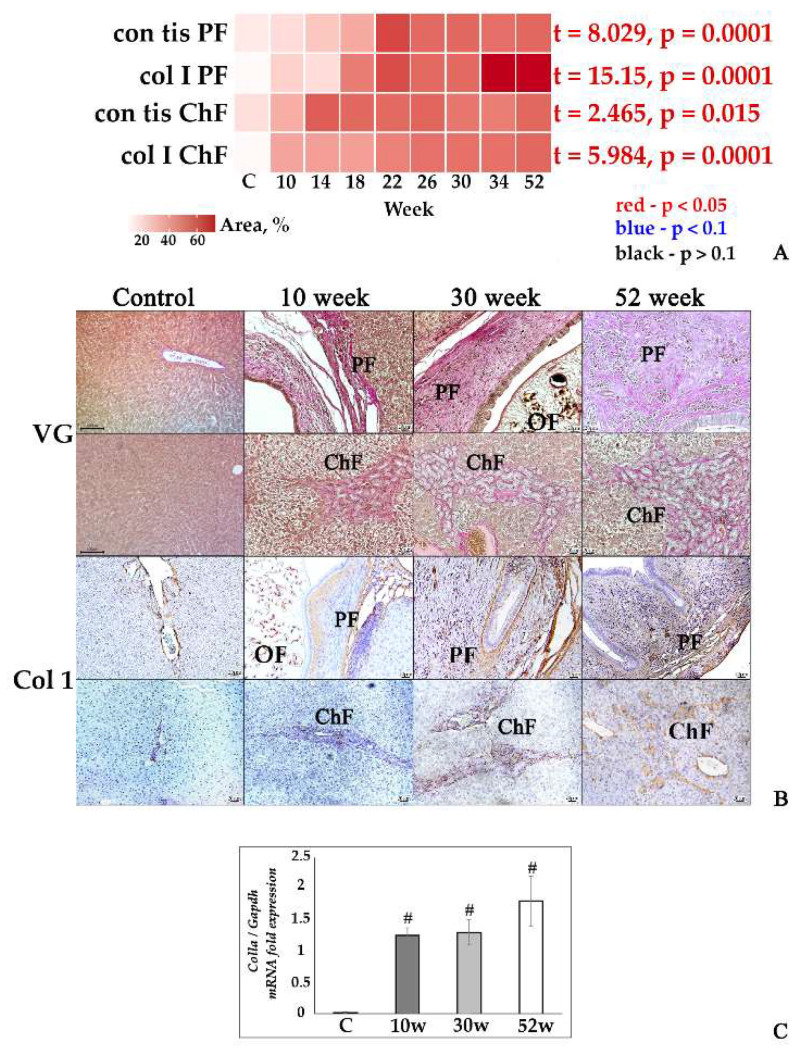
Fibrotic changes in the liver tissue of *O. felineus*-infected Syrian hamsters. (**A**) Heat map progression of periductal fibrosis (con_tis_PF) and cholangiofibrosis (con_tis_ChF), and the amount of collagen 1a+ fibers in the liver of infected animals (col_I_PF and col_I_ChF, respectively); (**B**) histopathological changes in Syrian hamster liver, Van Gieson staining (VG), and IHC analysis for collagen 1a (Col1); (**C**) *Col1a* gene was normalized to average *Gapdh* expression. C—control, 10 w, 30 w, 52 w—week p.i. Data are presented as mean ± SEM, # *p* ≤ 0.05, as compared to the control group.

**Figure 4:**
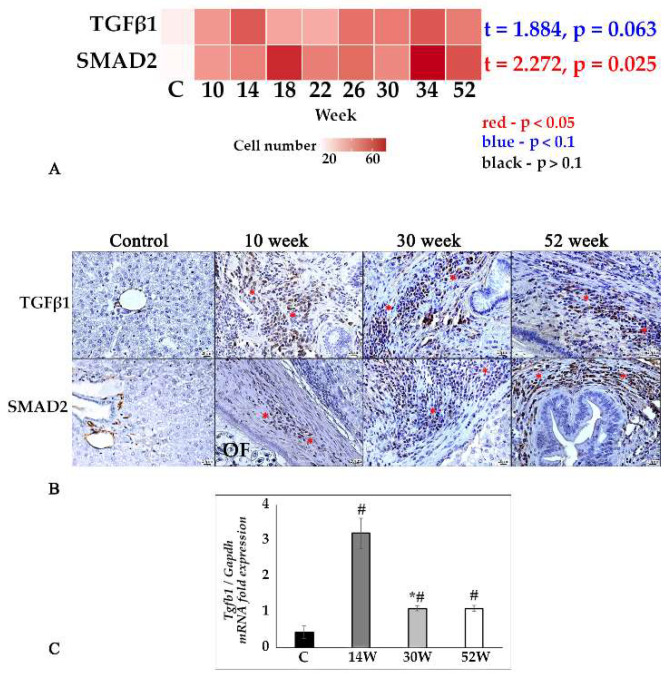
TGFβ1/SMAD2 signaling pathway in the liver tissue of *O. felineus*-infected Syrian hamsters. (**A**) Heat map of changes in TGF-β1- and SMAD2-positive cells in the course of infection; (**B**) TGF-β1- and SMAD2-positive cells (marked by an asterisk) in the dynamics of infection, IHC study, ×400 magnification; (**C**) *Tgfb1* gene was normalized to average *Gapdh* expression. C—control, 10 w, 30 w, 52 w—week p.i. Data are presented as mean ± SEM, # *p* ≤ 0.05, as compared to the control group, * *p* ≤ 0.05, as compared to the previous period of investigation.
