# Supplementary material for: Jagged-1/Notch Pathway and Key Transient Markers Involved in Biliary Fibrosis during Opisthorchis felineus Infection
Source: Trop Med Infect Dis. 2022 Nov 9;7(11):364. doi: 10.3390/tropicalmed7110364 (PMC9697314; doi:10.3390/tropicalmed7110364)
Supplement: Supplementary file 1 [file tropicalmed-07-00364-s001.zip › Supplementary material S2.pdf]

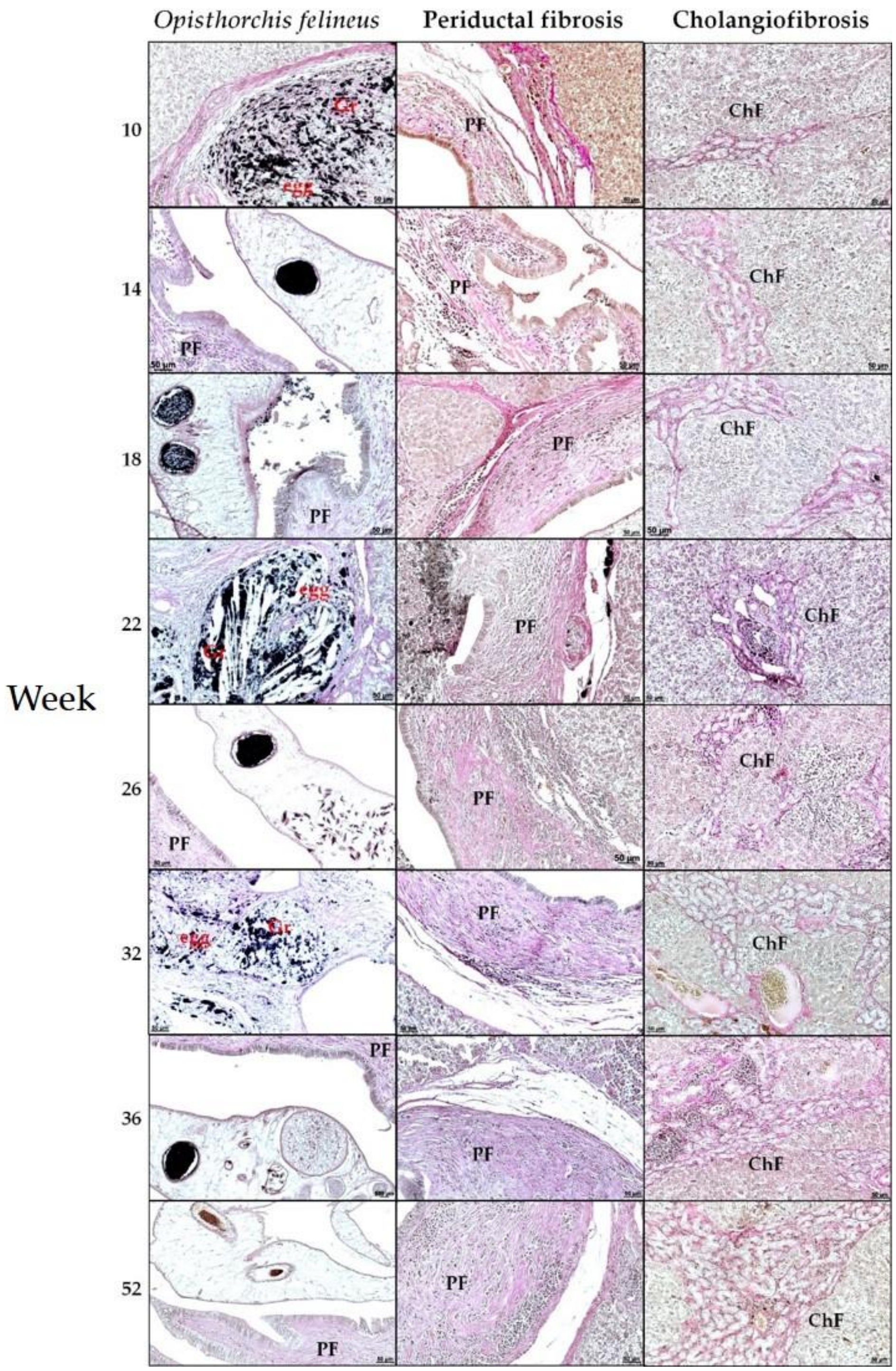

Figure S1: Histopathological changes in Syrian hamster liver, Van Gieson staining. OF – *Opisthorchis felineus*, Gr – granuloma, egg – *O. felineus* egg into granuloma, PF – periductal fibrosis, ChF – cholangiofibrosis. Magnification x200.

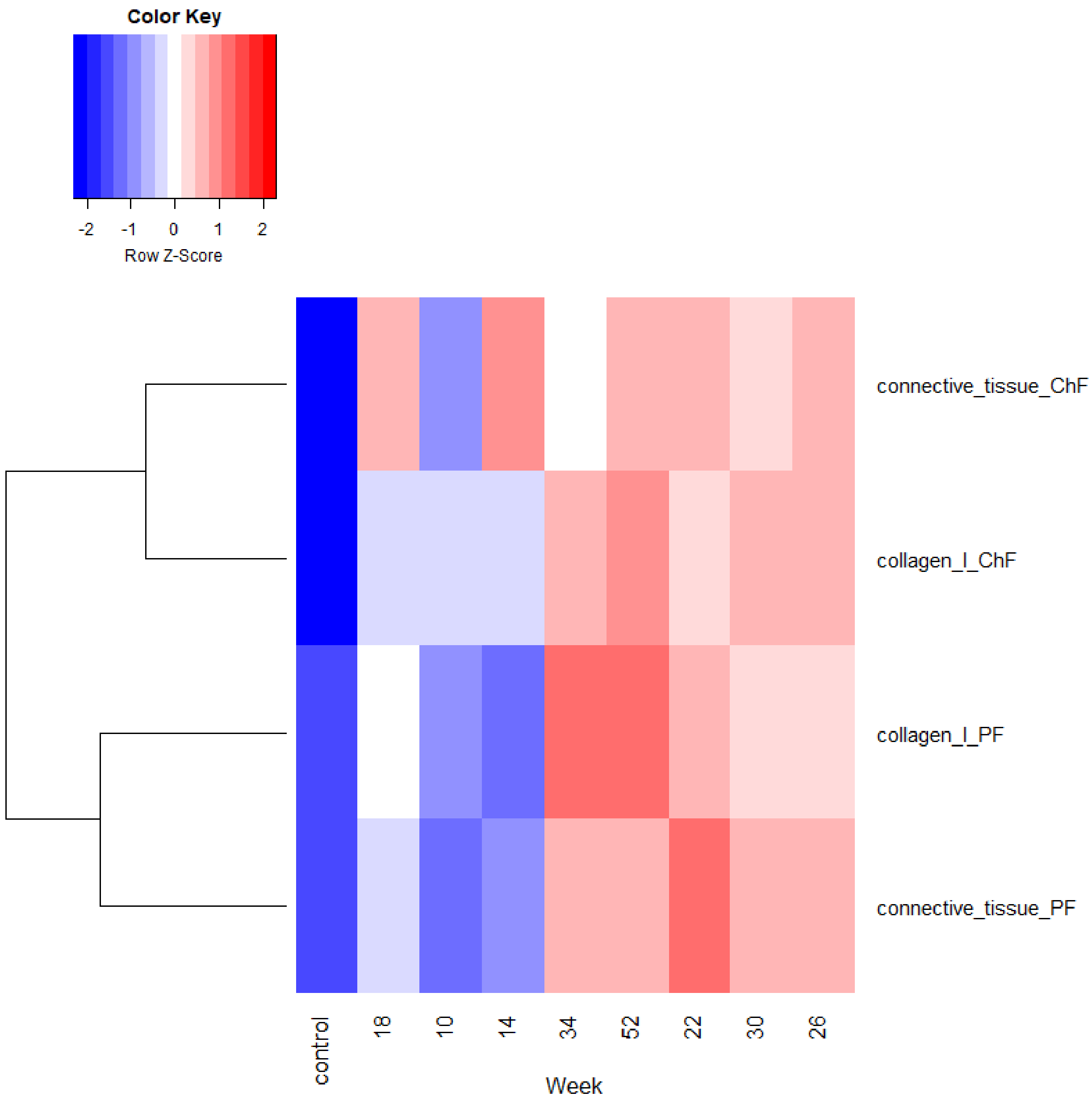

Figure S2: Clustering analysis of periductal and cholangiofibrosis with type I collagen.

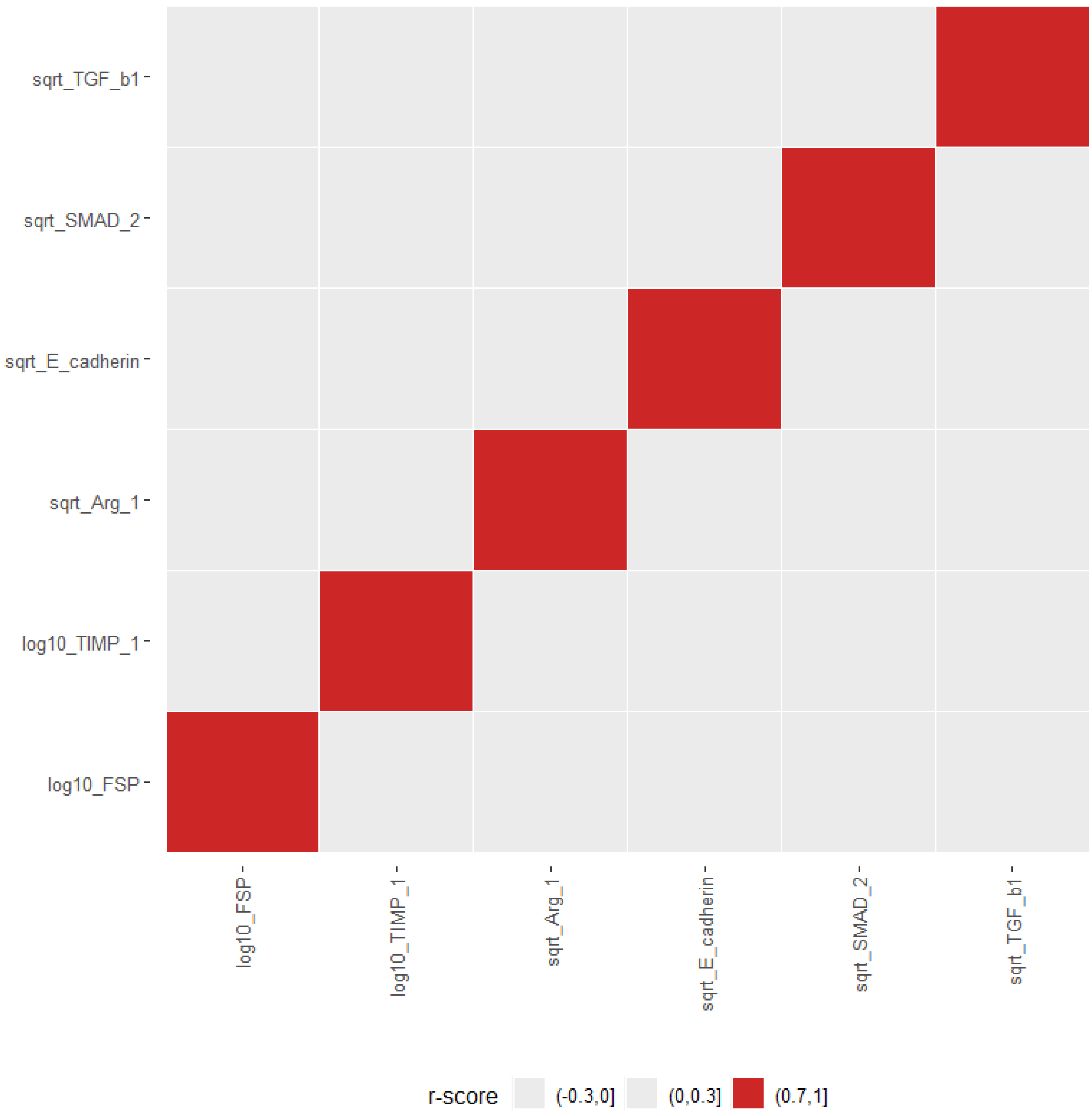

Figure S3: Calculation of parametric criterion Pearson correlation.

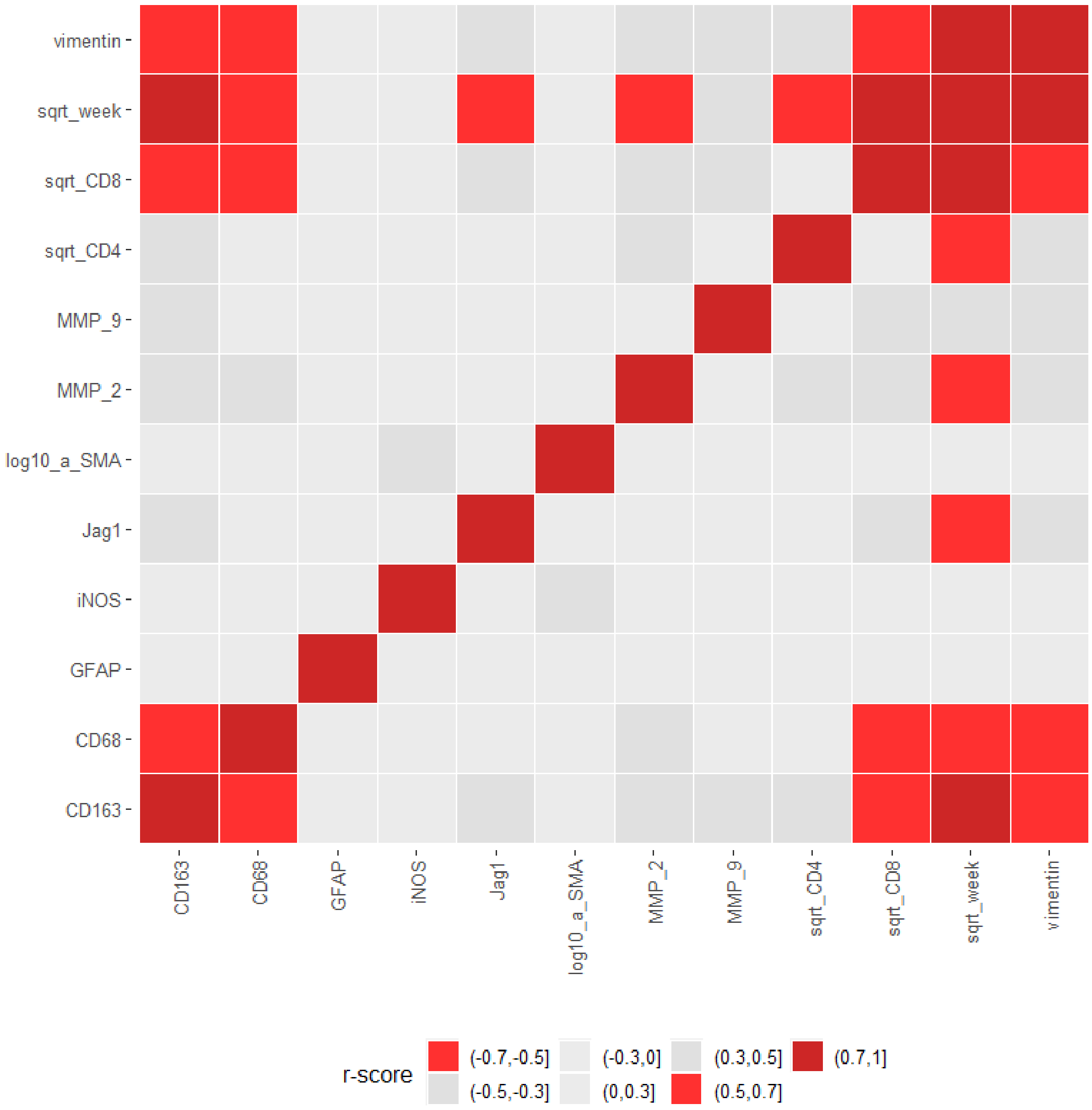

Figure S4: Calculation of non-parametric criterion Spearman correlation.
